# Using Metaphorical Nudges to Promote Mental Health Help-Seeking in Older Adults: Preliminary Results of an RCT

**DOI:** 10.1093/geroni/igaf122.3681

**Published:** 2025-12-31

**Authors:** Tianyin Liu, Ho Kan Liu

**Affiliations:** The Hong Kong Polytechnic University, Hong Kong, Hong Kong, China (People’s Republic); The Hong Kong Polytechnic University, Hong Kong, Hong Kong, China (People’s Republic)

## Abstract

Older adults are at an increased risk for mental health issues; however, their intention in seeking help is relatively low. To promote mental health help-seeking, nudging could be a viable strategy, but it has rarely been examined in older adults. This study evaluated the effects of pictorial metaphorical nudges (PMNs) and descriptive metaphorical nudges (DMNs) versus help-seeking information (Control) on promoting mental health help-seeking using a three-arm randomised controlled trial (RCT). The PMNs and DMNs were co-developed by older volunteers (N = 12) with experience in mental health services and delivered daily to participants via WhatsApp for two weeks. The primary outcomes were changes in subjective norms and help-seeking intention; secondary outcomes included perceived behavioral control, help-seeking attitude, perceived barriers to help-seeking, and mental health assessed by PHQ-9, GAD-7 and UCLA-3. In total, 280 participants (average age, 67.9 years; 214 females) were recruited from the community from February 1 to July 31, 2025, and they completed the baseline (T0), post-intervention (T1), and three-month follow-up (T2) questionnaires. Linear mixed models were used for data analysis. Results showed that PMN was more effective in improving subjective norms (β = 0.46, p <.05) and help-seeking attitudes (β = 0.28, p <.05) from T0 to T1 than Control; DMN and Control showed no significant difference. Data collection of more participants and at T2 is ongoing. Preliminary results suggest the feasibility and effects of metaphorical nudges in changing subjective norms and attitudes towards mental health help-seeking. Additionally, visual images may communicate complex information more effectively than text alone.

